# International benchmarking of specialty hospitals. A series of case studies on comprehensive cancer centres

**DOI:** 10.1186/1472-6963-10-253

**Published:** 2010-08-31

**Authors:** Wineke AM van Lent, Relinde D de Beer, Wim H van Harten

**Affiliations:** 1 Division of Psychosocial Research and Epidemiology, Netherlands Cancer Institute - Antoni van Leeuwenhoek Hospital, PO Box 902031006, BE Amsterdam, The Netherlands; 2 Ministry of Health, Welfare and Sport, The Hague, The Netherlands; 3 Department of Health Technology Services, Research School of Management and Governance, University of Twente, Enschede, The Netherlands

## Abstract

**Background:**

Benchmarking is one of the methods used in business that is applied to hospitals to improve the management of their operations. International comparison between hospitals can explain performance differences. As there is a trend towards specialization of hospitals, this study examines the benchmarking process and the success factors of benchmarking in international specialized cancer centres.

**Methods:**

Three independent international benchmarking studies on operations management in cancer centres were conducted. The first study included three comprehensive cancer centres (CCC), three chemotherapy day units (CDU) were involved in the second study and four radiotherapy departments were included in the final study. Per multiple case study a research protocol was used to structure the benchmarking process. After reviewing the multiple case studies, the resulting description was used to study the research objectives.

**Results:**

We adapted and evaluated existing benchmarking processes through formalizing stakeholder involvement and verifying the comparability of the partners. We also devised a framework to structure the indicators to produce a coherent indicator set and better improvement suggestions. Evaluating the feasibility of benchmarking as a tool to improve hospital processes led to mixed results. Case study 1 resulted in general recommendations for the organizations involved. In case study 2, the combination of benchmarking and lean management led in one CDU to a 24% increase in bed utilization and a 12% increase in productivity. Three radiotherapy departments of case study 3, were considering implementing the recommendations.

Additionally, success factors, such as a well-defined and small project scope, partner selection based on clear criteria, stakeholder involvement, simple and well-structured indicators, analysis of both the process and its results and, adapt the identified better working methods to the own setting, were found.

**Conclusions:**

The improved benchmarking process and the success factors can produce relevant input to improve the operations management of specialty hospitals.

## Background

Society is struggling with the challenge of cost containment in health care; costs are expected to grow considerably, mainly due to population ageing and the introduction of new technologies. Additionally, the workforce required to deliver the health care services is showing a relative decline. This has created growing interest in the performance of health services and the practices leading to excellent performance.

Research on operations management (OM) studies the production and delivery of products and services [1]. In order to improve their efficiency, hospitals are introducing OM practices, like benchmarking.

Benchmarking is defined as "*the search for- and implementation of best practices" *[2], it originated in the manufacturing industry and is now widely applied in healthcare. Gift and Mosel provide the following definition for healthcare: *"... benchmarking is the continual and collaborative discipline of measuring and comparing the results of key work processes with those of the best performers. It is learning how to adapt these best practices to achieve breakthrough process improvements and build healthier communities" *[3].

**Figure 1:**
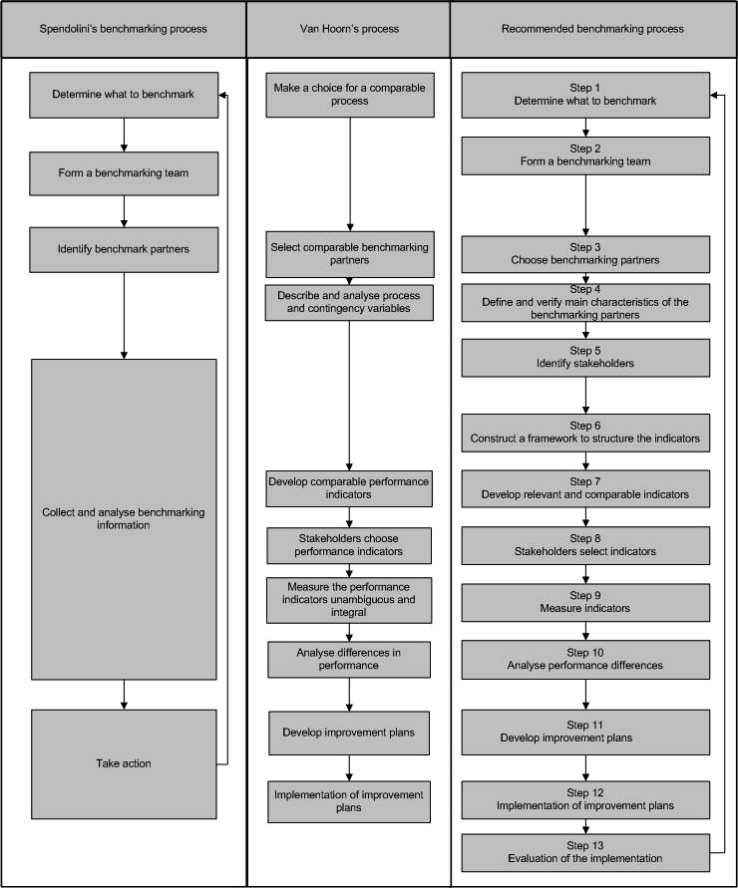
**Benchmarking processes compared**.

The literature presents numerous benchmarking processes [4,5]. Spendolini [5] compared 24 benchmarking processes and found four common characteristics in all of them, see Figure 1. Most benchmarking processes originated in manufacturing industries; therefore it is uncertain whether they are suitable for application to hospitals. Hospital services may be described as professional bureaucracies with characteristics like multiple stakeholders and possibly conflicting professional and business objectives. Van Hoorn et al [6] described a benchmarking process for healthcare, which is illustrated in Figure 1. This process [6] stressed the importance of creating project support and emphasized the need to assess the comparability of the organizations and the involvement of stakeholders in the development of indicators.

Health services research (HSR) applied benchmarking mainly to identify best practices for national health systems and treatments. The WHO World Health Report [7] concluded that although health status between countries was comparable, healthcare costs differed considerably. Nevertheless, the *"knowledge on the determinants of the health system performance, as distinct from understanding health status, remains very limited." *This conclusion underlines the possibility in understanding international practices as an instrument to improve healthcare performance. International benchmarking helps to explain for instance efficiency differences in hospitals and it supports hospitals to improve their processes.

Although international benchmarking on operations management may improve hospital processes, research on this subject is limited. It seems that so far most attention is given to the comparison of healthcare systems on a national level and to the development of indicators. The importance of indicator development is highlighted by Groene et al [8] who found 11 national indicator development projects in a systematic review.

This focus on indicators has also been adopted by healthcare agencies, like the National Health Service (NHS) in the UK, the Joint Commission on Accreditation of Healthcare Organizations (JCAHO) in the USA, and for-profit service providers. Under the term benchmarking, these organizations use indicators to publish hospital performance rankings, assuming that they foster competition and lead to the dissemination of best practices [9]. However, most rankings do not provide thorough insight into the organizational practices that led to the measured performance although this insight is required to improve healthcare processes, as they are often based on readily available administrative data sets [9].

We conclude that benchmarking as a tool to improve operations management in hospitals is not well described and possibly not well developed.

### Specialty hospitals and benchmarking

In order to become more efficient, healthcare is also showing a trend towards specialization of hospitals (or their units). Schneider et al [10] described specialty hospitals as hospitals "*that treat patients with specific medical conditions or those in need of specific medical or surgical procedures.*" The number of specialty hospitals is increasing [10-12]. Porter, Herzlinger and Christensen [13-15] suggested that specialization improves the performance, because it results in a better organization of processes, improved patient satisfaction, more cost-effective treatments and better outcomes. Most research involving specialty hospitals concentrated on the differences with general hospitals [10] whereas identifying optimal practices, especially regarding operations management, was seldom the topic of research.

Because specialty hospitals represent a trend and the opinions about the added value are divided, more insight into the benchmarking process in specialty hospitals could be useful to study differences in organization and performance and the identification of optimal work procedures.

Benchmarking of operations management in specialty hospitals has not been frequently examined. By the end of 2009, we could find only 23 papers in PubMed about operations management in specialty hospitals, 6 of them concerning cancer centres. About half of the 23 papers turned out to be a mismatch with the research topic. Most of the relevant papers appeared to be non-scientific, mentioned just a few outcomes, and emphasized the experiences of the project members. Only four publications reported on a competitive benchmark for specialty hospitals, but none described benchmarking in an international setting, nor did they focus on the benchmarking process or the success factors.

### Research questions

We conclude that international benchmarking as part of an approach to improve performance in specialty hospitals, has not been the subject of thorough research. Therefore, we address the following research questions:

1. What is the most suitable process for benchmarking operations management in international comprehensive cancer centres or departments (benchmarking process) to improve hospitals?

2. What are the success factors for international benchmarking in comprehensive cancer centres (success factors)?

## Methods

### Study design

International benchmarking with the objective to identify OM improvements in specialty hospitals is examined on the basis of three independent multiple case studies in comprehensive cancer centres. We used multiple case studies, because they are suitable for exploratory investigations and allow in-depth research. Each multiple case study consisted of international comprehensive cancer centres (CCC) or departments within a CCC, as these may be representative for specialty hospitals operating in an internationally competitive environment. A comprehensive cancer centre means a (partly) tertiary hospital specializing in the treatment of oncology patients, which is also involved in education and translational research.

Each multiple case study concerned a different hospital level: total hospital level, unit level and department level. Multiple case study 1 was limited to the comparison of operations management within CCCs. Three CCC's were included. In study 2 a small project scope was defined to enable to go through the complete benchmarking process, including the translation of more optimal working procedures and the evaluation of the implemented changes. Three chemotherapy day units (CDUs) were the cases for this study. In study 3 the scope was widened to a department, but the study was limited to the delivery of recommendations to the involved organizations. This study especially evaluated the involvement of internal stakeholders and the indicator development process. Radiotherapy departments were the cases of this study.

### Case selection

The purpose of the case studies was an international comparison with well-known, similar organizations to identify better working methods in operations management in specialty hospitals. The selected cases had to match the research objectives [16]. Since scarcely any objective data on best practices for OM in (specialty) hospitals were available it was impossible to select cases based on performance. Therefore convenient sampling was the most obvious way to obtain meaningful results.

Together with the stakeholders of the initiating centre the researchers developed inclusion criteria to verify to organizational comparability. Table 1 summarizes the three multiple case studies and their inclusion criteria. Patient characteristics were not verified in advance, since the mission and strategy of the comprehensive cancer centres suggested a similar case mix. Besides, better working methods could also be identified when patient characteristics differ.

**Table 1:** Overview of the case studies

*Multiple case study 1*	*Multiple case study 2*	*Multiple case study 3*
Operations management in Comprehensive Cancer Centres (CCC)	Operations management in Chemotherapy day units (CDU)	Operations management in Radiotherapy departments (RT)

Total organization	Unit level	Department level

Comprehensive cancer centres	Part of comprehensive cancer centres	Part of comprehensive cancer centres

	Only medication related treatments	Size: minimum of three linear accelerators

	1 with perceived high efficiency, 1 with > 50 beds	Strategy

		Patient case mix

3 European partners	3; 2 from Europe, 1 USA	4 European partners

Management approached potential participants, whenever participants fulfilled the criteria and agreed to participate, they were included. The organizations involved are presented anonymously in the text.

### Case study research protocol

To increase the reliability and validity of the case studies, the researchers developed a separate research protocol for each case study [17]. The protocols described the selection criteria for the hospitals involved, the benchmarking process, and the indicators. As case research protocols need to be tested [16], we piloted the research protocol in the initiating hospital. A distinction between HSR benchmarking and the approach taken in this paper was that our process focused on gaining insight into the organizational aspects, thus creating learning opportunities to improve performance. This research did not emphasize the development of extensively validated indicators or procedures to validate the comparability of organizations (for example on case mix).

In multiple case study 1 the benchmarking process was based on Spendolini's benchmarking process [5] (see Figure 1 and Table 2). Since this is a general model that has been based on benchmarking experiences in manufacturing industries, we scrutinized it, and when necessary adapted it to ensure a comprehensive and appropriate benchmarking approach. Table 2 describes the benchmarking process used in each case. The benchmarking process used in each multiple case study differs on details as the lessons learned were integrated in the next multiple case study.

**Table 2:** Benchmarking process employed per multiple case study

*Benchmark activity*	*Spendolini*	*Case 1: CCC*	*Case 2: CDU*	*Case 3: Radiotherapy*
1. Determine what to benchmark	**+**	**+**	**+**	**+**
2. Form a benchmarking team	**+**	**+**	**+**	**+**
3. Choose benchmarking partners	**+**	**+**	**+**	**+**
4. Define and verify the main characteristics of the partners	-	-	**+**	**+**
5. Identify stakeholders	-	-	-	**+**
6. Construct a framework to structure the indicators	-	**+**	**+**	**+**
7. Develop relevant and comparable indicators	-	**+**	**+**	**+**
8. Stakeholders select indicators	-	**+**	**+**	**+**
9. Measure the set of performance indicators	-	**+**	**+**	**+**
10. Analyse performance differences	**+**	**+**	**+**	**+**
11. Take action: results were presented in a report and recommendations were given	**+**	**+**	-	**+**
12. Develop improvement plans	-	-	**+**	**-**
13. Implement the improvement plans	-	-	**+**	**-**
14. Evaluation of the implementation	-	-	**+**	**-**

+ = used in benchmarking case study

- = not used in benchmarking case study

In multiple case study 1, we expanded Spendolini's benchmarking process [5] to include a framework that structured the indicators (Table 2, step 6), ensured comparability and covered all relevant aspects. We selected the EFQM (European Foundation for Quality Management) model because it considered strategic aspects, the processes and the outcomes. Another reason is that the EFQM model and its USA variant, the Malcolm Baldridge Quality Award (MBQA), are used in many hospitals [18]. Additionally, the step involving 'Collect and Analyse benchmarking information' was broken up into four phases: i) develop relevant and comparable indicators, step 7; ii) stakeholders select indicators, step 8; iii) measure the indicators, step 9; and iv) analyse performance differences, step 10. Finally, we separated the 'take action' into two phases: develop improvement plans (step 12) and implement improvement plans (step 13).

Table 2 shows the benchmarking process used in multiple case study 2. Compared to study 1, we added step 4. In this step we verified the comparability of the partners using the patient case mix (based on the ICD-9 coding system and treatment urgency) and the services delivered by the CDU.

In multiple case study 3 we additionally used input from a benchmarking process for healthcare developed by Van Hoorn et al (20), since this became available after study 2. This study emphasized the indicator development process, the involvement of internal stakeholders and the comparability of the results. Compared to case study 2, step 5 - identification of stakeholders - was added. In a stakeholder analysis [19-21] we identified cancer centre management, radiotherapy department management, radiation oncologists and clinical physicists as stakeholders. In collaboration with the stakeholders, the benchmarking team earmarked 'commitment' and 'shared ownership' for improvement suggestions.

At the start of each benchmark literature was searched for relevant indicators. Stakeholders of the initiating organization provided feedback, resulting in a reduced list of indicators. Although some only described a situation or condition, most indicators consisted of a numerator and a denominator. For example, the number of patients treated per linear accelerator per opening hour.

### Data collection methods

Industrial engineering and management students collected the data according to the research protocols. We used both qualitative and quantitative methods to collect data for each case. Quantitative data were retrieved from annual reports and requested from the administrative departments, whereas qualitative data were mainly collected by conducting semi-structured interviews during the site visits. In the CDU case, we also used direct observations to gain a better understanding of the processes that led to the results.

To increase the validity of the data, the outcomes of the indicators were presented to the contact persons of the relevant comprehensive cancer centres. Most quantitative indicators were collected from databases and were verified with the stakeholders; this process of triangulation increased the validity of the data [22].

### Data analysis

Per multiple case study the data for each indicator were compared. In cases of exceptional outcomes the persons who delivered the data were asked to comment on the differences. These explanations helped us to understand differences between the organizations. Besides comparing individual indicators, we took the total indicator set into consideration, because a good score on one indicator seemed to affect the performance on another indicator. For example a high utilization rate is related to longer access delays.

After reviewing each multiple case study the research team examined the feasibility, actual process and success factors of international benchmarking in comprehensive cancer centres.

## Results

Below we describe the findings to the research questions. Per question the results of the multiple case studies are presented. The indicators presented are examples of the indicators used to analyse the organizations involved.

### Question 1: benchmarking process

#### Multiple case study 1: comprehensive cancer centres (CCC)

The methods section already described the benchmarking process which is summarized in Table 2. The adapted benchmarking process of Spendolini [5] was workable, but adjustments might increase the generation of improvement suggestions regarding operations management. Although the CCCs were satisfied with the results, they commented that the results would not always be applied in change processes because they were uncertain that the same performance could be achieved in their setting because the processes might still not be sufficiently comparable.

The selected indicators distinguished between the total organization level, diagnostics, surgery, medication related treatments, radiotherapy and research. The results showed possibilities for improvements. For example, Table 3 shows that the percentages of staffing costs were comparable, but the percentage of non-medical support staff ranged between 24% (CCC2) and 15% (CCC1). CCC2 could thus learn from CCC1 to reduce the percentage of support staff. Regarding radiotherapy, CCC2 treated 53% more patients per linear accelerator, CCC3 could learn from CCC2 to improve their performance. Actually embarking on related improvement activities would however require further research.

**Table 3:** Examples of indicators for the benchmarking of CCC

*Indicator*	*Indicator type*	*CCC1*	*CCC2*	*CCC3*
Percentage staff costs on total costs (in %)	Organizational level	61	64	58

Percentage of supportive staff on total staff in full-time equivalents	Organizational level	15%	24%	Not available

Number of hospital beds (admission longer than 24 hours)	Department level	174	327	56, only beds for radiotherapy and internal medicine

Number of surgeries performed per OR	Department level	558	741	Not identifiable

Number of patients treated per linear accelerator	Department level	490*	480	313

Number of patient visits per CT scanner	Department level	7648	9047	Only for a specific treatment for one location available

Percentage of staff costs on total costs of radiology department	Department level	44	65	Not available

* = number of treatment series, not patients. 1 patient can receive multiple series.

#### Multiple case study 2: chemotherapy day units (CDU)

Since the CDUs found verification of their comparability useful, e.g. in respect of patient case mix treated and the services delivered, we included this as a new step in the benchmarking process (see Table 2, step 4). A self-made instrument was developed to test the comparability involved organizations. The case mix was examined with the ICD-9 coding system, the percentage of urgent patients and the duration of the treatments. The delivered services were examined based on the main techniques used for treatments. The patient case mix and services offered were similar, see Table 4[23].

**Table 4:** Examples of indicators for the benchmarking of CDU, see also [23]

*Items compared*	*CDU 1*	*CDU 2*	*CDU 3*
Patient case mix	23 out of 30	21-27 out of 30	23 out of 30

Services offered	28 out of 36	30 out of 36	28 out of 36

Total patient visits 2004	11.152	80.000	12.371

Estimated total patient visits 2005 in November	12.000	107.000	12.500

Indexed average number of patients treated per bed per month (not corrected for differences in opening hours)	44	77	100

Indexed average number of patient visits per month per total CDU staff	58	44	100

Indexed average number of patient visits per nurse per month	62	53	100

In Table 4 the estimated number of patient visits for 2005 shows that all CDUs were growing rapidly. CDU 3 clearly outperformed the others on the number of patients treated per bed and the number of patients treated per nurse or staff member, and provided possibly more optimal working methods for the planning procedure, reduction of non-value adding activities and nursing staff utilization.

The benchmark resulted in recommendations for improving patient planning and work procedures concerning resources (bed, nurses and medication) needed for a medical procedure. A multidisciplinary team implemented the recommendations by translating the lessons learned from CDU3 to CDU1. During this translation process, CDU1 also used lean management principles to obtain even better results. For more details about this improvement project, see Van Lent et al [23]. This resulted in a 24% growth in the number of patient visits, a 12% to 14% increase in staff productivity and an 80% reduction of overtime while the average expected treatment duration remained stable.

#### Multiple case study 3: radiotherapy departments (RT)

We further adapted the benchmarking process based on the work of Van Hoorn et al (20), on verifying the comparability of hospitals and developing indicators that achieve consensus among stakeholders. This suggested the researchers to examine the role of the stakeholders and the development of indicators more thoroughly. Just as in study 2, the tumour types of the patients (ICD-codes) were used to verify the comparability of the involved radiotherapy departments (Table 2, step 4). Since these data were not available for the departments we checked the comparability of the ICD-codes on a national level, assuming that the patients of radiotherapy centres reflected the national data on the use of international treatment protocols. The comparability was acceptable.

Compared to study 2, step 5 - identification of stakeholders with a stakeholder analysis [19-21] - was added. The improved benchmarking process (see Table 2) resulted in better acceptance of the indicators, although it proved difficult to obtain all the requested data.

The stakeholder analysis supported was also useful for the development of the indicators. Just as in the other studies an initial list of indicators was based on relevant literature. The stakeholders identified in step 5 provided feedback on the relevancy, measurability and comparability of the selected indicators. As a result indicators were removed, adapted and added. After the benchmark the indicator set was evaluated.

Table 5 presents examples of the benchmarked outcomes. For patient satisfaction and risk analysis, we measured whether the departments systematically applied the plan-do-check-act cycle to achieve improvements. None of the organizations performed all phases of the cycle; even the most optimal procedure did not keep track of the changes.

**Table 5:** Examples of indicators for the benchmarking of radiotherapy departments

*Examples of indicators*	*RT1*	*RT2*	*RT3*	*RT4*
Patient satisfaction, stage in PDCA cycle	Check-Act	Plan-Do	Do-Check	Plan- Do

Risk analysis, stage in PDCA cycle	Do- Check	Do-Check	Plan-Do	Plan-Do

Average impact points per publication and total publications	5.6 out of 297	2.3 out of 55	2.8 out of 68	Not available

Percentage of patients in clinical trials	4.4%	0.7%	10.7%	3.45%

Percentage of treatment planning with:	0	0	0	40
Simulator	91	98	77	56
CT	8	2	7	0
MRI	1	0	17	3
PET				

Patients treated per Linear Accelerator per standard opening hour	4.5	4.6	5.4	5.6

Number of hours of downtime for planned maintenance per Linear Accelerator	156	173	47,5	84


Our analysis revealed that radiotherapy centre 1 (RT1) seemed to have the most optimal working method for risk analysis, waiting times, patient satisfaction and scientific publications. RT3 and RT4 achieved better results regarding the Linear Accelerator utilization. Although the organizations involved accepted the results and recognized the improvement opportunities, they wanted more details before implementation because they did not have sufficient insight into the underlying organizational processes and the coherence between the indicators. A change in respect of one indicator (like a reduction of Linear Accelerator downtime) might affect the performance regarding another one (Linear Accelerator utilization).

RT1 started to work on their patient satisfaction and risk analysis score and a switch to measuring waiting time per tumour type instead of general waiting times is being considered. Furthermore, RT1 studied opportunities to reduce planned downtime during regular working hours. RT2 examined its inclusion rate for clinical trials and the information included in management reports. This should support them in making their status as a high-quality radiotherapy centre transparent. RT4 has been working on a system to register misses (part of the risk analysis) and it used the indicator outcomes to measure how many investments in staff and equipment are needed to remain a high-quality radiotherapy centre.

### Question 2: success factors for international benchmarking

#### Multiple case study 1: CCC

International benchmarking of a CCC on operations management is complex. Due to different reimbursement and accounting systems, the use of financial indicators was especially complex. Moreover, differences between external environments (mainly caused by government regulations) and the organizational choices resulted in difficulties with data availability.

Furthermore, policy affected the data directly and the organizational structure often determined the procedures for data collection and aggregation. The administrative organization of CCC3 was not yet capable of providing data for all activities as an identifiable unit on that level of organization because it shared resources with a general hospital. This problem was exacerbated because the CCC was in the middle of a merger and the data registration systems were not yet completely integrated. As the oncology surgeries could not be identified separately, it was impossible to verify the exact numbers. This case study used simple indicators, like patient-staff ratios or patient-resource ratios that could easily be collected.

An identifiable unit or department such as radiotherapy, radiology or a chemotherapy day unit seemed more suitable for benchmarking as this simplifies data collection. Radiology departments could be compared if referring policies are comparable. Specialized surgical departments seemed difficult to benchmark, due to problems with data availability, indicator definitions and the organizational embedding of the operating theatre.

#### Multiple case study 2: CDU

The small project scope together with the use of interviews and observations resulted in improved insight into the organizational principles that delivered the results. The benchmark made the partners aware that other organizations with similar problems were able to achieve better outcomes. This resulted in useful recommendations that have been implemented in CDU1. The management of CDU1 reported that the verification of the comparability had resulted in increased confidence in the identified improvement opportunities.

#### Multiple case study 3: RT

The results revealed that organization-specific characteristics influenced the outcomes because some departments, like radiotherapy, are quite dependent on technology (for example, most clinical trials require Linear Accelerators with special functions, like a cone beam). Thus, indicators measuring the percentage of patients included in clinical trials did not only reflect the organizational quality of the process, but rather the availability to scarce resources. This highlighted the importance of careful partner selection.

Comparable to multiple case study two, it is impossible to define a single most optimal working method for a department without considering its operational priorities. This should be taken into account whenever the team identifies a learning opportunity.

All indicators were measured over a one-year period. A discussion with the benchmarking partners revealed that some indicators were subject to large year-to-year variations. Examples are the average impact points per publication and the number of patients included in a clinical trial. Thus, measuring indicators over a one year period as done in this case study, does not always give a good impression of the performance.

## Discussion

Based on our results we present the following answers to the research questions.

1. What is the most suitable process for benchmarking operations management in international comprehensive cancer centres or departments (benchmarking process) to improve hospitals?

Figure 1 shows the recommended benchmarking process based on this study and compares it with the benchmarking processes of Spendolini [5] and Van Hoorn et al [6].

Compared to case study 3 (see Table 2) we have added translation of the improvement opportunities to the individual situation as a specification of step 12 (develop improvement plans) and the evaluation of the results and the benchmarking project (step 13). The project team has to establish consensus on the content of each step.

The results on the feasibility of benchmarking as a tool to improved hospital processes are mixed. Multiple case study 1 provided insight into the benchmarking process and gave indications for improvement opportunities. For study 2 we presented evidence of improvements. Although implementation was conducted together with lean management (see [23]), the benchmark enabled discussion about the working procedures and prevented a reinventing of the wheel because it gave direction to the improvements. Study 3 resulted in recommendations that are being considered for implementation. Altogether our conclusion confirms the work of De Korne et al [24] who concluded after an international benchmarking initiative of eye hospitals that it is possible but *"not so easy to compare performance in an international setting, especially if the goal is to quantify performance gaps or to identify best practices."*

2. *What are critical success factors for international benchmarking in comprehensive cancer centres?*

Table 6 summarizes the success factors and relates them to the steps described in the proposed benchmarking process in Figure 1.

**Table 6:** Success factors for the proposed benchmarking process

*Success factors for international benchmarking on operations management*	*Step in Figure 1*
Internal stakeholders should be convinced that others might have developed solutions for problems that can be translated to their own setting.	Step 1

Management must reserve sufficient resources for the total benchmark.	Step 1

Limit the scope to a well-defined problem.	Step 1

Define criteria to verify the comparability of the benchmarking partners based on the subject and the process.	Step 4

Construct a format that enables a structured comparison.	Step 6

Use both quantitative and qualitative data for measurement.	Step 7

Involve stakeholders to obtain consensus about the indicators, to provide information on data availability and reliability, and to assist in data collection.	Step 5,6,7,8,9

Keep indicators simple so that enough time can be spent on the analysis of the underlying processes.	Step 7,8,9,10

For those indicators showing a large annual variation in outcomes, measurement over a number of years should be considered.	Step 7,9,10

Adapt the identified better working methods, so that they comply with other practices in the organization.	Step 11

a. Before embarking on benchmarking to improve hospital (unit) performance, three additional conditions should be met. First, the internal stakeholders need to be convinced that others have developed solutions for problems that can be translated to their own setting (as in the CDU case), otherwise they might frustrate the project, by either questioning the validity of the outcomes or withholding implementation. Second, management must reserve sufficient resources (time and money) to execute the total process, including the development of improvement plans. Third, a manageable-sized project scope is required for a thorough analysis of the selected *process *and its results. The scope should be limited to a well-defined problem, for example capacity utilization. This can be found in an identifiable department or unit, but it can also be a small process that involves several departments.

b. The initiating organization should define criteria to verify the comparability of the benchmarking partners based on the subject and the process. Cases two and three showed the usefulness of the criteria for creating support for the benchmark and preventing comparability difficulties that were related to organization-specific characteristics. De Korne et al [24] concluded that a comparison with peers provided an incentive to professionals to change current practices.

Often information on hospitals is collected on a national level. Thus, specialty hospitals with few competitors in their own country will more frequently encounter a lack of data on comparable organizations. In these cases, possible partners should be contacted and screened on the inclusion criteria.

c. Stakeholder involvement is crucial for obtaining consensus about the indicators, providing information on data availability and reliability, and assisting in data collection.

d. Both quantitative and qualitative data should be used to determine the performance and construct a format that enables a structured comparison, such as the EFQM model.

e. Do not spend too much time on the reliability of the indicators, keep them simple so that enough time can be spent on the analysis of the underlying processes because this leads to improvement suggestions [4,24]. Qualitative indicators, observations and interviews should be used for this purpose. This is especially important in an international benchmark with a score for differences in and between organizations. A comparison of financial data has to be undertaken with care, especially in an international benchmark with different price levels and national reimbursement systems per country. Comparative purchasing power enables cost comparison [25]; however this is time consuming, whereas non-financial indicators sometimes achieve a good comparison within a shorter time frame.

f. For those indicators showing a large annual variation in outcomes, measurement over a number of years should be considered.

g. Adapt the identified better working methods, so that they comply with other practices in the organization.

To our knowledge, this is the first attempt that examined international benchmarking on operations management in (speciality) hospitals. The approach we followed made it possible to improve the structure of international benchmarking processes. This process in combination with the provided success factors may increase the chance that benchmarking results in improved operations management performance in specialty hospitals like comprehensive cancer centres.

A limitation is that our benchmarking process was only tested in three multiple case studies involving three to four cases. Involving larger series could be useful to further improve the validity of the benchmarking process. Furthermore, our multiple case studies were limited to cancer centres, but we presume that the benchmarking process is valid for other multidisciplinary specialty hospitals. Single specialty hospitals might be easier to compare. Further research is required to confirm this. As the benchmarking process seems more time consuming in an international setting as system differences add to the complexity, we suggest that the described process is useful for benchmarking in a national or regional setting provided the objective is to identify relevant operations aspects into sufficient depth.

To our knowledge there is no accepted guideline or norm describing a complete indicator set for comparing the operations management performance in hospitals or hospital departments. Per multiple case study we defined an initial list of indicators, based on relevant literature and stakeholder feedback. Stakeholders provided feedback on the relevancy, measurability and comparability. As a result indicators were removed, adapted and sometimes added. A limitation of this approach is that more emphasis could be laid on the methodological quality of the indicators. However, combining the benchmarking process with a thorough and detailed process of indicator development could further improve the benchmarking, but will prove to be complex and demanding. In this way generic indicator sets on operations management could become available.

The developed indicator sets enabled the assessment of the operations management of specialty hospitals and generated suggestions for improvement. Collecting and interpreting data, however, has to be done carefully and must be based on the total indicator set as there is not (yet) one single best method to organize processes. For example a good performance by one indicator (utilization rate) is often associated with a negative effect on another indicator (long waiting times).

A limitation of the sampling method is that it remains uncertain whether the best practices within the sector have actually been included. Because information on best practices was not available, we used personal management contacts to select presumed good working methods. As data availability and comparability seems more frequently a problem in an international context, we recommend the use of international benchmarking only if comparable organizations are not available within the same country.

Although Gift and Mosel [3] stated that benchmarking is a continuous process, the cases were only benchmarked once. Recurrent measuring seems only useful if different outcomes can be expected within short time frames, and the partners are ready for a long-term commitment.

## Conclusions

This study generated more insight into the process of international benchmarking as a tool to improve operations management in specialty hospitals. All multiple case studies provided areas for improvement and multiple case study 2 presented the results of a successful improvement project based on international benchmarking. The provided method and the success factors can be used in international benchmarking projects on operations management in speciality hospitals.

## Competing interests

Wim H van Harten is a member of the executive board of the Netherlands Cancer Institute - Antoni van Leeuwenhoek Hospital. Wineke AM van Lent is also employed by this organization as a PhD-student and advisor on patient logistics.

## Authors' contributions

WvL developed the research design and carried out case studies 1 and 2. She also drafted the manuscript. RdB improved the research design for case study 3 and conducted this case study. Furthermore, she contributed to revision of the manuscript. WvH was involved in the research design of all case studies, supervised them and contributed to the intellectual content of the manuscript. All authors read and approved the final manuscript.

## Pre-publication history

The pre-publication history for this paper can be accessed here:

http://www.biomedcentral.com/1472-6963/10/253/prepub
